# Spinocerebellar ataxias (SCAs) caused by common mutations

**DOI:** 10.1007/s10048-021-00662-5

**Published:** 2021-08-16

**Authors:** Ulrich Müller

**Affiliations:** Institute of Human Genetics, JLU-Gießen, Schlangenzahl 14, 35392 Giessen, Germany

**Keywords:** Spinocerebellar ataxias, Common mutations, Ca^2+^ homeostasis, Disease mechanisms

## Abstract

The term SCA refers to a phenotypically and genetically heterogeneous group of autosomal dominant spinocerebellar ataxias. Phenotypically they present as gait ataxia frequently in combination with dysarthria and oculomotor problems. Additional signs and symptoms are common and can include various pyramidal and extrapyramidal signs and intellectual impairment. Genetic causes of SCAs are either repeat expansions within disease genes or common mutations (point mutations, deletions, insertions etc.). Frequently the two types of mutations cause indistinguishable phenotypes (locus heterogeneity). This article focuses on SCAs caused by common mutations. It describes phenotype and genotype of the presently 27 types known and discusses the molecular pathogenesis in those 21 types where the disease gene has been identified. Apart from the dominant types, the article also summarizes findings in a variant caused by mutations in a mitochondrial gene. Possible common disease mechanisms are considered based on findings in the various SCAs described.

## Introduction

Spinocerebellar ataxias (SCAs) are a group of autosomal dominant ataxias characterized by cerebellar degeneration frequently in combination with brain stem atrophy. The major clinical signs are gait ataxia commonly associated with dysarthria and visual problems. Many additional signs and symptoms can occur including cognitive impairment, limb and trunk ataxia, tremor, rigidity, bradykinesia, dystonia, and hyperreflexia and may be pathognomonic in a few types of SCAs. Most SCAs, however, cannot be distinguished clinically and require genetic differentiation [1].

Currently about 50 different SCAs are distinguished defined either by their disease genes or by their chromosomal location if the disease gene is not yet known (https://neuromuscular.wustl.edu/ataxia/domatax.html). SCA disease genes/loci are found on most chromosomes. The mutations causing disease are frequently expansions of tandem repeats within the disease gene. These repeats are mostly composed of trinucleotides (mainly CAG; sometimes CTA/CTG) [2], but penta-and hexanucleotide repeat expansions can also cause SCAs such as SCAs 10, 37, and 36 [3–5]. Apart from repeat expansions, more common types of mutations (point mutations, deletions, insertions, duplications) underlie many different types of SCAs.

This article reviews the nature of non-repeat expansion mutations in specific SCAs. The phenotype of each SCA is described, followed by mapping and identification of the disease gene. The normal function of the disease gene is given and potential pathological mechanisms underlying disease are discussed. The few types of SCAs in which the disease gene has not yet been identified will also be covered. The article comprises 21 SCAs that are caused by common mutations. The description of the different types of SCAs is followed by a discussion of common features of these SCAs, possible common pathways of the gene products, and their potential interactions in different types of SCAs.

## SCA5

Main clinical findings of SCA5 are ataxia in combination with dysarthria and nystagmus. Age of onset varies widely. Ataxia is slowly progressive and mild. Patients usually remain ambulatory throughout life [6–8]. MRI analysis revealed marked global cerebellar atrophy [7].

Linkage analysis initially assigned the disease locus to the pericentromeric region of chromosome 11 [6]. Its location was subsequently narrowed to a 6.5-cM interval in chromosome region 11q13 [9].

The disease gene was identified as *SPTBN2* by the detection of mutations within this gene in patients from three families [10]. The mutations were two in frame deletions of 39 bp (c.1592_1630del/p.E532_M544del) and 15 bp (1886_1900del/ L629_R634delinsW), respectively, and a missense mutation (c.158 T > C/ p.L253P). To date about 20 different *SPTBN2* mutations have been described including small deletions and point mutations [11]. Among the latter, mutation c.1438C > T/p.R480W had occurred de novo in three unrelated cases [8, 12, 13] suggesting a mutational hot spot at this position of the gene. Mutation carriers present with childhood onset ataxia and dysarthria in addition to other symptoms such as developmental delay. Cerebellar hypoplasia can be detected by MRI at an early age (~ 2 years). These cases suggest a specific correlation between the R480W mutation and the resulting severe phenotype [8, 12, 13].

*SPTBN2* codes for β-III-spectrin and is highly expressed in Purkinje cells and the cerebral cortex [10; https://www.proteinatlas.org/ENSG00000173898-SPTBN2/tissue]. β-III-spectrin is associated with the Golgi apparatus and cytoplasmic vesicles [14], binds to actin via ARP1 (actin related protein) [15], stabilizes the glutamate reporter EAAT4, and regulates glutamate signaling [10].

Mutations of *SPTBN2* disturb other plasma membrane proteins such as glutamate receptor δ2 (GluRd2) that are regulated by β-III-spectrin, in particular excitatory amino acid transporter 4 (EAAT4), a post-synaptic glutamate transporter at Purkinje cell synapses [10]. Ikeda et al. speculate that disturbed expression and loss of EAAT4 and GluRd2 at the plasma membrane “could lead to glutamate signaling abnormalities that, over time, could cause Purkinje cell death in SCA5” [10]. Studying the pathological mechanism of one specific *SPTBN2* mutation (p.L253P), Avery et al. conclude that the mutation disturbs high affinity binding of β-III-spectrin to actin and mediates neurotoxicity by impacting the dynamics of the spectrin-actin network and thus of microfilaments [16].

## SCA11

SCA11 is a relatively benign, late-onset, and slowly progressive ataxia that can be associated with eye signs (jerky pursuit, nystagmus), and pyramidal features (increased muscular tonus, increased reflexes, and Babinski sign). MRI documents cerebellar atrophy [17, 18].

A disease locus was initially assigned to a 7.6-cM interval on chromosome 15 (15q14-q21.3) by linkage analysis in a family with autosomal dominant ataxia [17] and further narrowed to a region of 5.6 cM in 15q15-q21 in a large 8 generation family [18].

A candidate gene approach revealed two different mutations in the gene *TTBK2* that encodes tau tubulin kinase 2 [18]. The mutations were a 1-bp insertion (c.1329insA/p.R444T) and a 2-bp deletion (c.1284_1285delAG/p.E429Dfs). Both mutations cause a frame shift that results in a premature stop codon and in truncation of tau tubulin kinase 2. An additional small deletion mutation of *TTBK2* (c.1306_1307delGA/p.D435fs448X) was described that also results in truncation of tau tubulin kinase 2 [19]. This indicates that frame shift mutations caused by small (1 or 2 bp) deletions or insertions in *TTBK2* are a common cause of SCA11 [20].

TTBK2 kinase phosphorylates microtubule-associated protein tau, microtubule-associated protein 2 (MAP2), and β-tubulin [20, 21]. In addition, it appears to phosphorylate the centriolar distal appendage protein CEP164 and atypical kinesin KIF2A located at the + tips of microtubules [22]. Interaction with microtubular proteins is mediated via the long C-terminus of TTBK2 kinase [23]. One additional function of TTBK2 kinase is the initiation of assembly of primary cilia during embryogenesis [24]. TTBK2 localizes to primary cilia [25].

Truncated tau tubulin kinase 2 in SCA11 encoded by mutated alleles in SCA11 appears to interfere with normal function of wild-type TTBK2 via a mild dominant negative effect. This in turn results in insufficient regulation of above proteins which are important in normal development and function of microtubuli in various cell types including cerebellar Purkinje cells [20]. Apart from the cerebellum, TTBK2 protein is expressed in multiple organs and at significantly higher level in some (e.g., bronchus, lung, smooth muscle, testis, Fallopian tube) (www.proteinatlas.org/ENSG00000128881-TTBK2/tissue). It requires further investigations to explain the apparently specific cerebellar effects of *TTBK2* mutations. Possibly the assumed disease mechanism, i.e., interference of truncated TTBK2 with the wildtype protein, does not occur in all tissues or results in only moderately reduced function.

## SCA13

SCA13 is a relatively mild form of ataxia. Both severity and age of onset vary considerably even within the same family. One or several signs and symptoms can occur in addition to ataxia, such as nystagmus, mild to moderate intellectual disability, myoclonic jerks, dysphagia, bradykinesia, and increased tendon reflexes.

[26–28]. Life expectancy of patients is not significantly reduced as compared to unaffected controls. Moderate cerebellar and pontine atrophy was shown by MRI in two patients [28].

The disease locus of SCA13 was initially assigned to a 11.4-cM interval on chromosome 19 (19q13.3-q13.4) [26] and later refined to ~ 4 cM at the same chromosome location [29].

Studying patients of two families, Waters et al. detected two missense mutations (c.1554G > A/p.R420H; c.1639C > A/p.F448L) in the gene *KCNC3* which is located within the critical interval on chromosome 19 [30].The two mutations detected alter the function of KCNC3 in a Xenopus laevis expression system. Since the first description of *KCNC3*, additional point mutations were detected in a sporadic [8] and in several familial cases [e.g., 28, 31].

*KCNC3* encodes potassium voltage-gated channel subfamily C member 3 (Kv3.3). Voltage-gated potassium channels are important in the rapid repolarization of fast firing neurons of the brain. Kv3.3 binds to actin and stabilizes the cortical actin network [32]. Mutations of *KCNC3* disturb regulation of the neuronal actin network thus disturbing normal regulation of duration and frequency of action potentials. This in turn interferes with reorganization and function of voltage-gated Ca^2+^ channels and of Ca^2+^ homeostasis which is required for survival and normal performance of Purkinje cells, i.e., their role in motor function [30, 32, 33].

## SCA14

SCA14 is a slowly progressive ataxia associated with dysarthria and nystagmus. Additional signs and symptoms can include myoclonus, tremor, dystonia, depression, and cognitive impairment [e.g., 34, 35, 36]. Pronounced cerebellar atrophy has been documented by magnetic resonance imaging (MRI) [37].

Linkage analysis assigned the disease locus to region q13.4-qter of chromosome 19 [34]. The disease gene, *PRKCG*, within this region was subsequently identified by the discovery of three mutations in three different families [37, 38]. The mutations within *PRKCG* (c.301C > T/ p.H101Y; c.355 T > C/p.S119P; c.383G > A/p.S119P) are missense mutations that affect a highly conserved residue in C1, i.e., the cysteine-rich region of the protein PKCγ. This results in cytoplasmic mislocation and aggregation of PKCγ. Aggregation appears to result in reduced degradation of PKCγ resulting in increased substrate phosphorylation [39]. Additional mutations in *PRKCG* included point mutations and small deletions [40, 41].

**Fig. 1:**
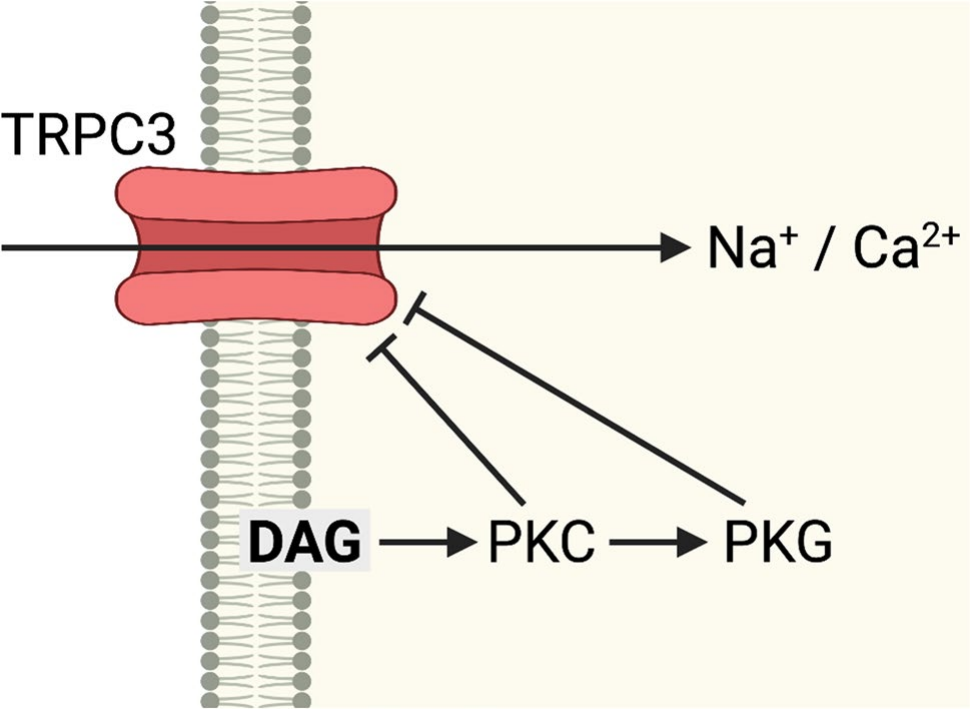
Negative regulation of TRPC3 by PKC at the postsynaptic membrane (modified from [118]). For details see text. TRPC3, short transient receptor potential channel 3; DAG, *diacylglycerol*; *PKC*, *protein kinase c*; PKG, protein kinase g

*PRKCG* encodes protein kinase Cγ (PKCγ), a member of a family of serine- and threonine-specific protein kinases. PKCγ is activated by Ca^2+^ and by diacylglycerol and is exclusively expressed in neurons of brain and spinal cord. One of the many functions of PKC is phosphorylation of GRIN1/NMDAR receptors (GRIN1/NMDAR encode Glutamate [NMDA] receptor subunit 1) that play an important role in synaptogenesis, synaptic plasticity, excitotoxicity, and mental functions. The kinase is also involved in innervation of Purkinje cells during cerebellar development [42–44]. Furthermore, it appears to have an inhibitory effect on TRPC3 channel activity (Fig. 1) at the postsynaptic membrane thus linking SCA14 and SCA41 that is caused by *TRPC3* mutations (see below and Fig. 1).

The effect of *PRKCG* mutations in SCA14 may be explained by increased cellular Ca^2+^ influx and a resulting disturbance of Ca^2+^ homeostasis. This in turn interferes with normal synaptic differentiation resulting in neuronal degeneration and/or abnormal function of Purkinje cells during cerebellar differentiation.

## SCA15

SCA15 (formerly SCA15/16) is a slowly progressive gait and limb ataxia frequently associated with ocular disturbances (nystagmus, saccadic eye movements), dysarthria, and dysphagia [45, 46]. Mild cognitive disturbance can occur. While gait ataxia appears to be mild in most cases, in one family, patients were walker or wheelchair-bound by age 15–17 [47]. Age of onset varies widely in SCA15. MRI revealed cerebellar atrophy [46] that mainly affected the vermis [47]. Brain stem was not affected.

Linkage analysis assigned the disease locus to 3p26.1-p25.3 [48, 49]. One gene, i.e., *itpr1*, was shown to cause an autosomal recessive movement disorder in mice similar to human ataxias. The human version this gene (*ITPR1*) maps to the critical interval on chromosome 3 that contains the SCA15 locus. Mutation analysis of this gene revealed deletions in 3 patients thus demonstrating that *ITPR1* is the disease gene in SCA15. The deletions involve a large portion of the region containing *ITPR1* which result in reduced expression of *ITPR1* [50]. Additional mutations detected in *ITPR1* include point mutations and deletions of various sizes that involved the entire gene [e.g., 51, 52]. Sequence variants are common within *ITPR1* (https://databases.lovd.nl/shared/variants/ITPR1), and discrimination between benign and malign sequence changes may be difficult.

*ITPR1* encodes Inositol 1,4,5-trisphosphate receptor type 1. This intracellular receptor is located at intracellular membranes such as the endoplasmic reticulum (ER) and mediates calcium release after stimulation by inositol 1,4,5-trisphosphate. (https://www.uniprot.org/uniprot/Q14643#function; http://www.ensembl.org/Homo_sapiens/Gene/Ontologies/biological_process?g=ENSG00000150995;r=3:4493345-4847506). The abnormal calcium levels caused by *ITPR1* mutations are cytotoxic, particularly in cerebellar Purkinje cells, and reveal a potential pathological mechanism in SCA15 [53, 54].

## SCA18

This disorder, now referred to as SCA18, was detected in one large family [55]. The authors referred to this slowly progressive syndrome as sensorimotor neuropathy with ataxia (SMNA). Signs and symptoms include gait ataxia, sensory loss, dysmetria, dysarthria, nystagmus, and weakness of arms and legs. Signs and symptoms varied greatly between affected persons of this family. Linkage analysis assigned the disease locus to 7q22-q32. Despite identifying a candidate gene, it has not been convincingly shown that this is indeed the disease gene in SCA18 [56].

## SCA19

SCA19 presents as a comparatively mild, slowly progressive ataxia frequently associated with myoclonus, tremor, dysarthria, and oculomotor abnormalities such as nystagmus [57, 58]. The age of onset varies greatly both within and between families. Severe phenotypes of SCA19 occur and can present with Parkinsonism, epilepsy, and pronounced cognitive problems [59].

Neuropathologic abnormalities include degeneration of portions of the cerebellum, particularly of the vermis and of Purkinje cells [60].

The disease locus has been assigned to a comparatively large interval on chromosome 1 (1p21-q21) in a Dutch family [61, 62].

Exome sequencing of the critical interval in patients identified the disease gene as *KCND3* by the detection of a small deletion (c.679_681delTTC, p.F227del) and of point mutations (e.g., c.1054 A > C/p.T352P; c.1119 G > A/p.M373I; c.1169 G > A/p.S390N) which were located in the evolutionarily highly conserved channel pore and the S6 transmembrane domain [58, 60]. (Note that this ataxia was referred to as SCA22 by the Chinese investigators and as SCA19 by the Dutch group before the discovery of *KCND3* mutations revealed that SCA19 and SCA22 are identical).

*KCND3* encodes potassium ionic channel Kv4.3, a fast inactivating, transient, A-type potassium channel. Although originally thought to be mainly expressed in heart and brain [63, 64], Kv4.3 has now been detected in most tissues at comparable levels (www.proteinatlas.org/ENSG00000171385-KCND3/tissue). Mutations of *KCND3* result in disturbance of ion channel and of voltage-gated potassium channel activity. It is not known, however, how this could lead to cerebellar atrophy. One hypothesis assumes that abnormal channel function disturbs calcium homeostasis. Abnormal calcium levels are cyototoxic and may mainly affect Purkinje cells [60].

## SCA20

This ataxia is mainly clinically defined since the disease gene has not yet been identified. SCA20 presents as slowly progressive ataxia and dysarthria which can precede ataxia. Associated symptoms can include palatal tremor, dysphonia, and dysarthria. Calcification of the dentate nucleus is detected by CT. MRI reveals cerebellar atrophy but no involvement of the brain stem [65].

A 260-kb duplication of 11p13-q11 is thought to cause the disease [66] but the specific gene(s) underlying SCA20 have not yet been identified.

## SCA21

SCA21 is a slowly progressive ataxia with cognitive impairment. Onset is commonly during childhood. However, onset during adulthood has also been observed. Frequent additional signs are limb ataxia, dysarthria, akinesia, and tremor. Cerebellar atrophy is detected by MRI [67, 68].

Linkage analysis assigned the SCA21 locus to 1p36.33-p36.32 [67]. Whole exome sequencing revealed several mutations within the gene *TMEM240* in index cases of seven families, six missense (c.509C > T/p.P170L; c.239C > T/p.T80M; c.346C > T/p.R116C; c.445G > A/p.E149K; c.511C > T/p.R171W; c.509C > T/p.P170L), and one stop codon mutation (c.489C > G/p.Y163*). All mutations altered highly conserved amino acid residues in TMEM240 [67].

*TMEM240* codes for transmembrane protein 240. Its expression is high in the brain, particularly in the frontal cortex and in the cerebellum [69]. It is a component of the plasma membrane and has also been observed in the mouse brain synapse membrane [70]. The function of TMEM240 is currently not understood. However, given its high expression in the frontal cortex (https://www.genecards.org/cgi-bin/carddisp.pl?gene=TMEM240) and its association with synaptic membranes, mutations interfere with some functions of the forebrain and might explain cognitive impairment in SCA28. Its high expression in cerebellum is also consistent with cerebellar atrophy which might be caused by dysfunction of membranes in particular of synapses and subsequent cell death.

## SCA23

SCA23 is an extremely rare late-onset, slowly progressive gait and limb ataxia that is frequently associated with dysarthria, dysmetria, slow saccadic eye movements, positive Babinski reflex, and impaired proprioception. Neuropathological findings in one patient revealed neuronal degeneration and loss in the Purkinje cell layer, dentate nuclei, and inferior olives. Furthermore, demyelination was observed in lateral and posterior columns of the spinal cord [71].

Linkage analysis in one family assigned the disease locus to chromosome region 20p13-p12.3) [71].

Analysis of the critical interval of the SCA23 locus detected four missense mutations in the gene *PDYN* (prodynorphin) in affected members of two Dutch families and in two apparently sporadic cases [72]. The mutations were c.414G > T/p.R138S, c.632 T > C/p.L211S), c.634C > T/p.R212W, and c.643C > T/p.R215C.

*PDYN* encodes prodynorphin that undergoes proteolysis to form the secreted opioid peptides beta-neoendorphin, dynorphins A and B, leu-enkephalin, rimorphin, and leumorphin. The dynorphins A and C are mainly located in Purkinje cells [72]. DynorphinA (DynA) is neurotoxic and appears to induce neurodegeneration via glutamate receptors and acid-sensing ion channels [73, 74]. Significantly, of above SCA mutations, p.R212W and p.R215C result in increased DynA levels as compared to controls. Consistent with these findings is the observation in vitro of increased loss of striatal neurons following exposure to p.R212W and p.R215C dynA [72]. Taken together, these observations facilitate a better understanding of the pathological mechanisms that underlie SCA23.

## SCA25

SCA25 has been described in one family. It is an autosomal dominant syndrome transmitted with reduced penetrance. Major clinical signs and symptoms are cerebellar ataxia and prominent sensory neuropathy frequently associated with altered eye movements (nystagmus and slow eye movement) and hypotonia. SCA25 is clinically highly heterogeneous. Age of onset is mainly during childhood with a wide range of onset (17 months to 39 years). Global cerebellar atrophy was shown by MRI. Linkage analysis assigned the disease locus to chromosome 2p21-p13. Presently no disease gene has been identified [75].

## SCA26

SCA26 is a late-onset syndrome characterized by ataxia frequently associated with irregular eye movements and dysarthria. Cerebellar ataxia was documented by MRI. Age of onset is during adulthood. The disorder has presently been detected in one large family only. The disease locus was assigned to chromosome 19p13.3 [76].

Deep sequencing of the critical region in 19p13.3 identified a C > A transversion that segregated with affected persons of the pedigree. This base change lies within exon 12 of the gene *EEF2* and results in the exchange of a proline by a histidine (p.P596H) of eEF2. The mutation cosegregated in affected members of the family described by Yu et al. [76]. Given that there are no additional families with this condition, the assumption of *EEF2* being the disease gene mainly relies on the location of the mutation in an evolutionarily highly conserved region of eEF2.

*EEF2* is expressed in all tissues. It codes for eukaryotic translation elongation factor 2 (eEF2) that facilitates the GTP-dependent translocation of peptidy-tRNA from the A—(aminoacyl-) site to the P—(peptidyl-)-site of the ribosome (the A-site is the first binding site for peptidyl-tRNA in the ribosome; the P-site is the second) [77, 78]. Presently it is not known how this mutation specifically causes cerebellar atrophy. Hekman et al. [79] studied the disease mechanism in yeast at the position of EF corresponding to P596H of human eEF2. They showed that this mutation increases frameshifting during translation. They speculate that cerebellar Purkinje cells are particularly susceptible to this amino acid change in eEF2 as compared to other tissues. The resulting disturbed protein synthesis is thought to cause cell death that eventually leads to cerebellar atrophy.

## SCA27

SCA27 is characterized by slowly progressive cerebellar ataxia, early-onset tremor, orofacial dyskinesia frequently in association with ocular problems (nystagmus, dysmetric saccades, strabismus), psychiatric symptoms, and cognitive deficits. Disease onset is in late childhood/early adulthood [80]. Cerebellar atrophy and degeneration of basal ganglia are detected by MRI in some but not in all patients [80].

Linkage analysis assigned the disease locus to chromosome region 13q34, and a candidate gene approach revealed a mutation c.434 T > C/p.F145S in the fibroblast growth factor 14 (*FGF14*) gene [80]. The findings of additional *FGF14* mutations (e.g., a 1 bp deletion c.487delA/p.D163fsX12 [81] and a missense mutation (c.529A > T/p.K177X) [82]) in autosomal dominant ataxias confirm that *FGF14* is the disease gene in SCA27.

FGF14 is encoded by the *FGF14* gene. It is mainly expressed in the brain particularly in the cerebellum (https://www.proteinatlas.org/ENSG00000102466-FGF14/tissue). Apart from its growth factor activity, FGF14 plays a role in — among others — nervous system development [83], regulation of voltage-gated calcium channel activity, regulation of synaptic plasticity, and synaptic vesicle recycling (http://www.ensembl.org/Homo_sapiens/Gene/Ontologies/biological_process?g=ENSG00000102466;r=13:101710804-102402457). Expression of *FGF14* is reduced in heterozygous carriers of a missense mutation, and as a result FGF14-mediated functions appear to be disturbed. Disruption of some of these functions such as Ca^2+^ homeostasis or synaptic differentiation and function could explain ataxia.

## SCA28

Clinical features of SCA28 are gait ataxia and unbalanced standing frequently in combination with ocular problems (nystagmus, slow saccades, ophthalmoplegia, ptosis), increased tendon reflexes in legs, and dysarthria [84, 85]. MRI analysis in patients shows cerebellar atrophy.

Linkage analysis assigned the disease locus to the pericentromeric region of chromosome 18 (18p11.22-q11.2) [84]. Sequencing of candidate genes within this region identified missense mutations in the gene *AFG3L2* in patients of five unrelated families (c.2071G > A/p.Q691K; c.2021delCCinsTA/p.S674L; c.2081C > A/p.A694E; c.2105G > A/p.R702Q; c.1296A > C/p.N432T) [86].

*AFG3L2* encodes mitochondrial AFG3 Like Matrix AAA Peptidase Subunit 2, a component of mitochondrial-AAA metalloprotease. It is an ATP-dependent protease involved in multiple biological processes such as mitochondrial protein processing, calcium import into the mitochondrion, and nerve development (https://www.ensembl.org/Homo_sapiens/Gene/Ontologies/biological_process?g=ENSG00000141385;r=18:12328944-12377227). *AFG3L2* is highly and selectively expressed in Purkinje cells [86]. The mutation affects the multiple functions of the protein. This is likely to result in a decrease of cellular energy production and eventually in Purkinje cell death and cerebellar atrophy. According to Di Bella et al., mutations of *AFG3L2* cause cerebellar atrophy by interference with its physiological role in the “mitochondrial protein quality control machinery” [86].

## SCA30

SCA30 is a relatively pure, slowly progressive ataxia that has been described in one family [87]. Apart from gait ataxia and dysarthria, ocular problems such as nystagmus were found in some family members. Disease onset in this family was during adulthood. Linkage analysis assigned the disease locus to 4q34.3-q35.1. Although the disease gene has not been identified, the authors discuss *ODZ3* as a candidate gene. *ODZ3* is also expressed in the brain and encodes the transmembrane protein teneurin3.

## SCA32

SCA32 is a cerebellar ataxia that has been described in one Chinese family [88]. Family members had ataxia and mental impairment (those with disease onset < 40 years), and all affected males of the family had azoospermia. Cerebellar atrophy was shown by MRI. The disease locus was mapped to 7q32-q33. A disease gene has not been identified. This type of ataxia has only been described in a meeting abstract; a complete, peer-reviewed paper has not been published. Therefore, the significance of this ataxia remains unclear until it is described in independent families.

## SCA34

Ataxia with erythrokeratodermia is referred to as SCA34 [89]. The skin problems disappear by age 24 or can be completely absent in some families [90, 91]. Onset of ataxia is during adulthood. Additional signs can be nystagmus and reduced tendon reflexes. Retinitis pigmentosa was detected in one family. SCA34 is mild to moderate and slowly progressive. MRI revealed atrophy of cerebellum, pons, and cortex. The disease locus was mapped to 6p12.3-q16.2 [89]. A candidate gene approach and exome sequencing identified a mutation in the gene *ELOVL4* (c.504G > C/p.L168F). Another mutation within *ELOVL4* (c.736 T > G/ W246G) was discovered in a Japanese family [90].

*ELOVL4* encodes ELOVL Fatty Acid Elongase 4, a membrane-bound protein of the ER involved in the biosynthesis of fatty acids by catalyzing the first step in very long-chain fatty acid elongation (https://www.uniprot.org/uniprot/Q9GZR5#function). Presently, it is not known how *ELOVL4* mutations might cause cerebellar atrophy and skin defects. One theory is based on the assumption that altered ELOVL Fatty Acid Elongase 4 disturbs integrity of the ER membrane. This might result in abnormal Ca^2+^ transport and thus disturb Ca^2+^ homeostasis that eventually results in Purkinje cell degeneration.

## SCA35

Signs and symptoms of SCA35 are late onset, slowly progressive gait and limb ataxia, ocular problems (dysmetria, nystagmus, occasionally slow saccades), dysarthria, and problems associated with upper motor neurons (pseudobulbar palsy, and brisk tendon reflexes). Disease onset is during adulthood (mainly 5th decade). Cerebellar atrophy was shown by MRI in patients [92, 93]. Linkage analysis in a large Chinese family assigned the disease locus to chromosome 20p13-p12.2 [92]. Exome sequencing identified the disease gene as *TGM6*. Missense mutations and small deletions were found in several unrelated Chinese families (e.g., c.1550 T > G/p.L517W [91]; c.1528G > C/D510H [92]; c.1722_1724delAGA/p.E574del [94]).

*TGM6* codes for transglutaminase 6. Glutaminases are Ca^2+^-dependent enzymes that catalyze cross-linking of proteins and attachment of polyamines to proteins. *TGM6* is expressed in many tissues including the brain (https://www.genecards.org/cgi-bin/carddisp.pl?gene=TGM6). It is presently not known how mutations of *TGM6* might cause cerebellar atrophy.

## SCA38

SCA38 is characterized by truncal and gait ataxia and nystagmus. The syndrome is slowly progressive. Age of onset is during adulthood. Cerebellar atrophy in patients was documented by MRI [95].

Linkage analysis assigned the SCA38 locus to a 56.2-Mb interval on chromosome 6p22.2-q14.1. Sequencing of the critical region identified missense mutations (c.214C > G/p.Leu72Val and c.689G > T/p.Gly230Val) in the gene *ELOVL5* in patients of two unrelated families [95].

*ELOVL5* encodes ELOVL fatty acid elongase 5. This enzyme is involved in fatty acid metabolism by catalyzing elongation of long-chain polyunsaturated fatty acids. *ELOVL5* is highly expressed in endocrine tissues and in the cerebellum and other regions of the brain (https://www.proteinatlas.org/ENSG00000188211-NCR3LG1/tissue). While the wild-type enzyme appears to be mainly located in the ER, the mutant enzyme was mainly found in the Golgi apparatus and much less in the ER [95]. Presently, these findings do not contribute to a convincing understanding of the pathological processes that eventually result in cerebellar atrophy. A more general interpretation of the role of mutated ELOVL fatty acid elongase is perturbation of lipid metabolism that might interfere with membrane formation and function and as a result in cell death. A possible explanation of cell death is altered Ca^2+^ permeability caused by membrane alterations that might alter Ca^2+^ homeostasis. Significantly, apart from the ER, other organelles including the Golgi apparatus are also actively involved in Ca^2+^ uptake and release (albeit by different mechanism than those regulating Ca^2+^ concentrations in the ER) [96].

## SCA 39

This type of SCA has been described in one family [97]. One patient of this family was examined in detail. He displayed cerebellar ataxia, spasticity of lower limbs, dysmetria of upper limbs, dysarthria, ocular problems (strabismus, saccades, horizontal gaze palsy), and mild mental retardation. Age of onset was during childhood; the patient was wheel-chair bound by age 41. MRI showed cerebellar atrophy and hyperintensity of periventricular white matter.

Mode of inheritance of the disease was compatible with autosomal dominant. SNP genotyping revealed a 7.5 Mb duplication on the long arm of chromosome 11 (q21-22.3). The duplication that segregated in the family contains 44 genes. The role of one or of several of these genes in the development of the observed phenotype is not known [97].

## SCA40

SCA40 was identified in one family [98]. Two patients were examined in detail. The patients shared adulthood disease onset and slow progression. They were wheelchair bound 17 and 18 years after disease onset owing to severe ataxia. Major signs and symptoms included gait ataxia, ocular dysmetria, brisk reflexes, and dysarthria. Both patients did not share all these signs. MRI showed pontocerebellar atrophy. Mode of inheritance appeared to be autosomal dominant. Performing whole exome sequencing in four affected and two unaffected members of the family, Tsoi et al. [98] identified a point mutation (c.1391G > A/p.R464H) in the gene *CCDC88C*.

*CCDC88C* encodes Coiled-Coil Domain Containing 88C (alternatively designated as KIAA1509). The mutation detected in the patients might result in a gain of function. It causes hyperphosphorylation of JNK (c-Jun N-terminal kinase) in cells overexpressing mutant *CCDC88C*. This in turn appears to activate caspase-3 which might result in apoptosis. More families need to be identified with mutations in CCDC88C to establish a role of mutations in this gene in the development of spinocerebellar ataxia.

## SCA41

The phenotype of SCA41 was described in one patient whose family history was incomplete [99]. Therefore, this patient could be a sporadic case. The main sign in this patient was gait ataxia. MRI revealed mild atrophy of the cerebellar vermis.

Performing whole exome sequencing, the authors detected a potentially pathogenic sequence change in the gene *TRPC3* (Chr4:122824185G > A/ p.R762H). *TRPC3* encodes transient receptor potential cation channel subfamily C member 3. *TRPC3* can be induced to form a non-selective channel permeable to Ca^2+^ and other cations. It may be induced by a phosphatidylinositol second messenger system and can also be activated by depletion of intracellular calcium. Intriguingly, mutations of the murine homologue of *TRPC3*, *trpc3*, cause ataxia in the mouse [100]. The mutation may disturb Ca^2+^ homeostasis by interfering with permeability of this cation. This together with the location of the R762H mutation within an evolutionarily highly conserved domain of the protein, makes *TRPC3* a convincing candidate for SCA41. However, more cases have to be documented before *TRPC3* can be established beyond doubt as the disease gene in SCA41.

## SCA43

SCA43 was discovered in one family in whom late onset sensorimotor axonal polyneuropathy segregated as an autosomal dominant trait [101]. Of the six affected members of the family, five also had cerebellar ataxia. Moderate vermis atrophy was shown by MRI in one patient.

A combination of linkage analysis and whole exome sequencing revealed a heterozygous transition G > A in the gene *MME* on chromosome 3q25.2 (p.C143Y) that was neither detected in unaffected members of the family nor present in dbSNP and EVS (exome variant server; https://evs.gs.washington.edu/EVS/) thus suggesting that this base change is a pathogenic mutation. The *MME* gene codes for membrane metalloendopeptidase (Neprilysin). This peptidase is expressed in many tissues and inactivates various proteins including Met- and Leu-enkephalins, angiotensin 1–9, amyloid beta (Aβ) and atrial natriuretic factor (ANF) and brain natriuretic factor (BNP(1–32) (https://www.genecards.org/cgi-bin/carddisp.pl?gene=MME). The pathological mechanism of heterozygous *MME* mutations in SCA43 is unknown [101]. Clearly more families with MME mutations need to be identified to establish that SCA43 does indeed play a role in the development of spinocerebellar ataxia.

## SCA45

SCA45 was discovered in a large family with autosomal dominant ataxia. Main signs of SCA45 are late-onset limb and gait ataxia, dysarthria, and nystagmus. Brain MRI revealed atrophy of the cerebellar vermis [102].

Whole exome sequencing revealed a mutation in the gene *FAT2* in affected members of this family (c.10758G > C/K3586N) and another *FAT2* mutation in an apparently sporadic case. (c.10946G > A/p.R3649Q) [102]. More recently another *FAT2* missense mutation (c.10906 T > G/p.Y3636D) was discovered in two siblings [103].

*FAT2* encodes the integral membrane protein FAT atypical cadherin 2 that functions as a cell adhesion protein and appears to bind Ca^2+^ (https://www.genecards.org/cgi-bin/carddisp.pl?gene=FAT2).

*FAT2* is expressed in cerebellar granule cells. It appears to play an important role in the development of the cerebellum by modulating the extracellular space surrounding parallel fibers [104]. Thus, mutations of *FAT2* might interfere with normal postnatal cerebellar development. A pathological mechanism might be disturbance of cellular Ca^2+^ equilibrium by abnormal binding of calcium ions.

## SCA46

Nibbeling et al. performed whole genome sequencing in various SCA families and detected a mutation in the gene *PLD3* in one family [102]. Signs in patients of this family included gait and limb ataxia, dysarthria, nystagmus, and sensory axonal neuropathy. Onset was during adulthood.

*PLD3* encodes phospholipase D family member 3. The gene is highly expressed in the brain, including the cerebellum. PLD3 catalyzes hydrolysis of cell membrane phospholipids. The mutation found in affected members of this family was c.923 T > C/p.L308P and resulted in a decrease of PLD3 activity. Cellular location, expression, and stability of the enzyme were not affected. The function of mutPLD3 in the development of SCA43 is presently unknown. *PLD3* was shown, however, to be functionally associated with established SCA genes that may be involved in the function of synapses [102]. However, more families need to be found that will confirm mutations of *PLD3* as a cause of spinocerebellar ataxia.

## SCA 47

SCA47 has been detected in two unrelated girls and in one family. Ataxia was passed on as an autosomal dominant trait with reduced penetrance in the family [105]. The disorder was sporadic in the two affected girls. Disease onset was during early childhood in the girls and during adulthood in affected family members. Disease was progressive in both the two sporadic cases and in affected adults of the family. Of the 9 affected members of the family, all had developmental delay but only 6 ataxia. Intellectual disability was noted in 7, and seizures occurred in 3. The girls had ataxia and developmental delay that affected speech and motor skills. In one girl first signs were severe seizures at the age of 5 months followed by ataxia, spasticity, intellectual disability, visual problems, and epileptic encephalopathy. MRI documented atrophy of the cerebellar vermis of patients.

A microdeletion within chromosome region 1p35.2 that included *PUM1* had been previously documented in the less severely affected girl [106]. Furthermore, mice with mutations in the gene *Pum1* develop ataxia. These findings prompted Gennario et al. to investigate above patients for mutations in this gene [107]. Exome sequencing revealed a heterozygous de novo missense mutation in the more severely affected sporadic childhood-onset case (g31406186 G > A/p.R1147W) and in affected family members (p.T1035S, transcript NM_001020658.1).

*PUM1* codes for Pumilio RNA Binding Family Member 1. It is a RNA-binding protein and is involved in the regulation of translation of specific mRNAs and thus plays a role in multiple cellular processes including regulation of neuronal function. It binds to ATXN1 (ataxin1) encoding mRNAs. By downregulating ataxin1 PUM1 helps to maintain ATXN1 levels. The missense mutations within *PUM1* result in destabilization of PUM1. This causes reduced binding to ATXN1 and upregulation of translation of ATXN1 mRNA. Increased ATXN1 levels result in their intracellular precipitation [107]. ATXN1 is mutated in SCA1 (CAG expansion), a mutation that also results in precipitation of the mutated protein, yet owing to polyglutamine [108]. Significantly, the phenotype of SCA47 is comparable to that of SCA1.

## SCA48

SCA48 presents as adult-onset cerebellar cognitive-affective syndrome (CCAS) and/or late-onset SCA. Signs include gait ataxia, dysarthria, cognitive decline, depression, and anxiety . Occasionally movement abnormalities (tremor, chorea etc.) are observed [109]. MRI in patients revealed atrophy of the posterior region of the cerebellar vermis [110]. SCA48 has now been described in several families from different countries [e.g., 100, 111].

The disease gene in the family of Genis et al. [110] was identified by whole exome sequencing and linkage analysis. The authors identified a frameshift mutation in the gene *STUB1*, located on chromosome 16p13.3 (c.823_824delCT/p.L275Dfs*16).

*STUB1* encodes STIP1 homology and U-box containing protein 1. Among other functions, it “collaborates with ATXN3 in the degradation of misfolded chaperone substrates” (https://www.genecards.org/cgi-bin/carddisp.pl?gene=STUB1). Given that a CAG expansion in ATXN3 results in SCA3/MJD, this “collaboration” of STIP1 with ATXN3 links SCA48 via its disease gene *STUB1* to another form of SCA (SCA3). This is similar to SCA47 that is linked to SCA1 via PUM1 that regulates ataxin 1. Although the exact cellular roles of ataxin 1 and 3 are not well understood yet, both seem to have nuclear functions: Ataxin1 is a chromatin binding factor and ataxin 3 binds to histones and regulates transcription.

## Adult-onset spinocerebellar ataxia, mitochondrial

In a considerable proportion of adult onset spinocerebellar ataxias (~ 50%), the underlying cause has not been established [112]. A relatively high percentage (> 5%) of undiagnosed familial as well as sporadic adult-onset spinocerebellar cases is caused by mutations in the mitochondrial gene *MT-ATP6* [113]. Therefore, this mitochondrial type of spinocerebellar ataxia is included in this article. The phenotype of ataxias caused by *MT-ATP6* mutations is clinically not different from late-onset autosomal forms of ataxia. Patients present with adult-onset gait ataxia, dysarthria, and ocular abnormalities such as nystagmus, sometimes in association with tremor and cognitive problems. Yet mutations in this gene can cause a wide spectrum of disease such as infantile-onset Leigh syndrome, NARP syndrome (neuropathy, ataxia, and retinitis pigmentosa), Charcot-Marie-Tooth syndrome, and adult onset spinocerebellar ataxia [114].

*MT-ATP6* encodes a subunit of mitochondrial ATP synthase, i.e., complex V of the mitochondrial respiratory chain. Mutations can interfere with mitochondrial ATP synthesis, increase the potential of the mitochondrial membrane, or interfere with ATP hydrolysis. Currently it is not known why the phenotype of patients with *MT-ATP6* mutations varies widely [114].

A lack of cellular energy production, i.e., reduced or absent synthesis of ATP, can explain many disease phenotypes. The reason for their wide phenotypic heterogeneity, however, remains poorly understood. A common finding in mitochondrial disorders, i.e., a varying degree of heteroplasmy (percentage of mutant vs. wildtype carrying mitochondria), does not account for the pronounced phenotypic heterogeneity of carriers of *MT-ATP6* mutations.

## Conclusions

**Fig. 2:**
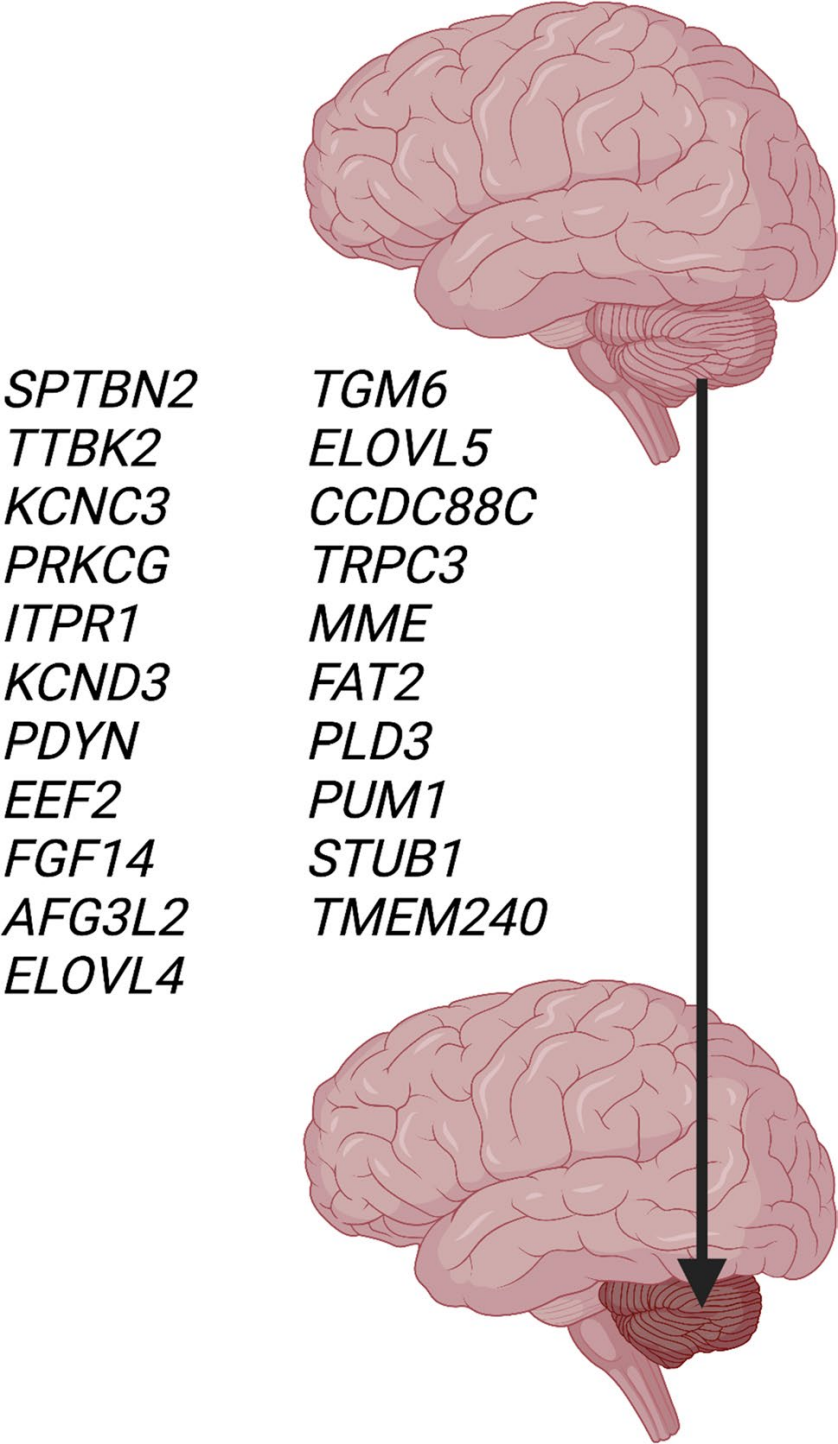
Twenty-one genes that cause spinocerebellar degeneration when mutated (see text and Table 1)

This review includes 28 SCAs that are not caused by repeat expansions. Of the SCAs reviewed, 27 are transmitted as autosomal dominant and one as mitochondrial trait. Several types of SCAs have been described in one family only (SCAs 18, 20, 25, 26, 30, 32, 39, 40, 41,43, 46). The disease gene is known in 21 autosomal dominant types (Table 1) and in the mitochondrial SCA. Mutations in these genes include point mutations and small deletions. Several disease genes have been observed in one family only and include *EEF2* (SCA26), *CCDC88C* (SCA 40), *TRPC3* (SCA 41), *MME* (SCA43), and *PLD3* (SCA46) and require confirmation in additional families. In 6 types of SCAs (SCAs 18, 20, 25, 30, 32, 39), the disease gene has not yet been identified but the disease locus has been chromosomally mapped. Two of these SCAs (20, 39) carry chromosomal duplications at 11p13-q11 and 11q21-22.3, respectively, that contain many genes. The specific disease-causing gene(s) have not been discovered. Figure 2 depicts the 21 nuclear genes that when mutated cause cerebellar atrophy.

**Table 1:** Genes mutated in SCAs, and their tissue expression, cellular compartments, and functions that are affected by the mutations

Gene	Gene product	Tissue expressionRNA and/or protein	Mutation affecting
*KCNC3*	Potassium voltage—gated channel, subfamily C, Kv.3.3	High in brain, less in other tissues	Ca^2+^homeostasis, microfilament cytoskeleton
*ITPR1*	Inositol 1,4,5 triphosphate receptor 1	Highest in brain	Ca^2+^ homeostasis
*TRPC3*	Transient receptor potential cation channel, subfamily 3	Brain, multiple other tissues	Ca^2+^homeostasis
*KCND3*	Potassium voltage-gated channel, subfamily D, Kv4.3	Brain, other tissues, similar level	Ca^2+^homeostasis
*FGF14*	Fibroblast growth factor 14	Highest in brain	Ca^2+^homeostasis??
*FAT2*	Fat atypical cadherin 2	High brain, less few other tissues	Ca^2+^homeostasis??
*SPTBN2*	Beta-III spectrin	Highest brain, few other tissues	Micro-filament stability,cytoskeleton
*TTBK2*	Tau tubulin kinase 2	Many tissues	Microtubuli, cytoskeleton
*PRKCG*	Protein kinase Cγ (PKCγ)	Neuron-specific	Synapses, Purkinje cells, Ca^2+^ homeostasis
*TMEM240*	Transmembrane protein 240	High in brain,Less in other tissues	Plasma membraneSynapses
*PLD3*	Phospholipase D family member 3		Synapses?
*ELOVL5*	ELOVL fatty acid elongase 5	Brain, higher level in other tissues	Membrane ER calcium homeostasis ?
*ELOVL4*	ELVOVL fatty acid elongase 4	Brain, higher skin, endocrine, lymphoid tissue	membrane ER calcium homeostasis
*PUM1*	Pumilio RNA Binding Family Member 1	Ubiquitous	Ataxin 1
*STUB1*	STIP1 homology and U-box containing protein 1	Ubiquitous	Ataxin 3
CCDC88C	Coiled-Coil Domain Containing 88C	Ubiquitous	Cell survival (induction apoptosis)
*AFG3L2*	Mitochondrial AFG3 Like Matrix AAA Peptidase Subunit 2	Ubiquitous	Mitochondrion, Purkinje cells
*EEF2*	Eukaryotic translation elongation factor	Ubiquitous	Translation
*PDYN*	Prodynorphin	Brain-specific	Striatal neurons
TGM6	Transglutaminase 6	Ubiquitous	??
*MME*	Membrane metalloendopeptidase (Neprilysin)	many tissues, very low brain	??

The SCAs discussed here differ from those caused by tandem repeat expansions, most commonly of (CAG)_n_, in two major ways. (1) They lack anticipation, i.e., earlier disease onset and more severe signs and symptoms in subsequent generations caused by repeat expansions, and (2) in contrast to SCAs caused by (CAG)_n_ expansions, in the SCAs discussed here, no cellular (cytoplasmic and/or nuclear) aggregation of toxic polyglutamines [115] is found that can explain neuronal death.

**Fig. 3:**
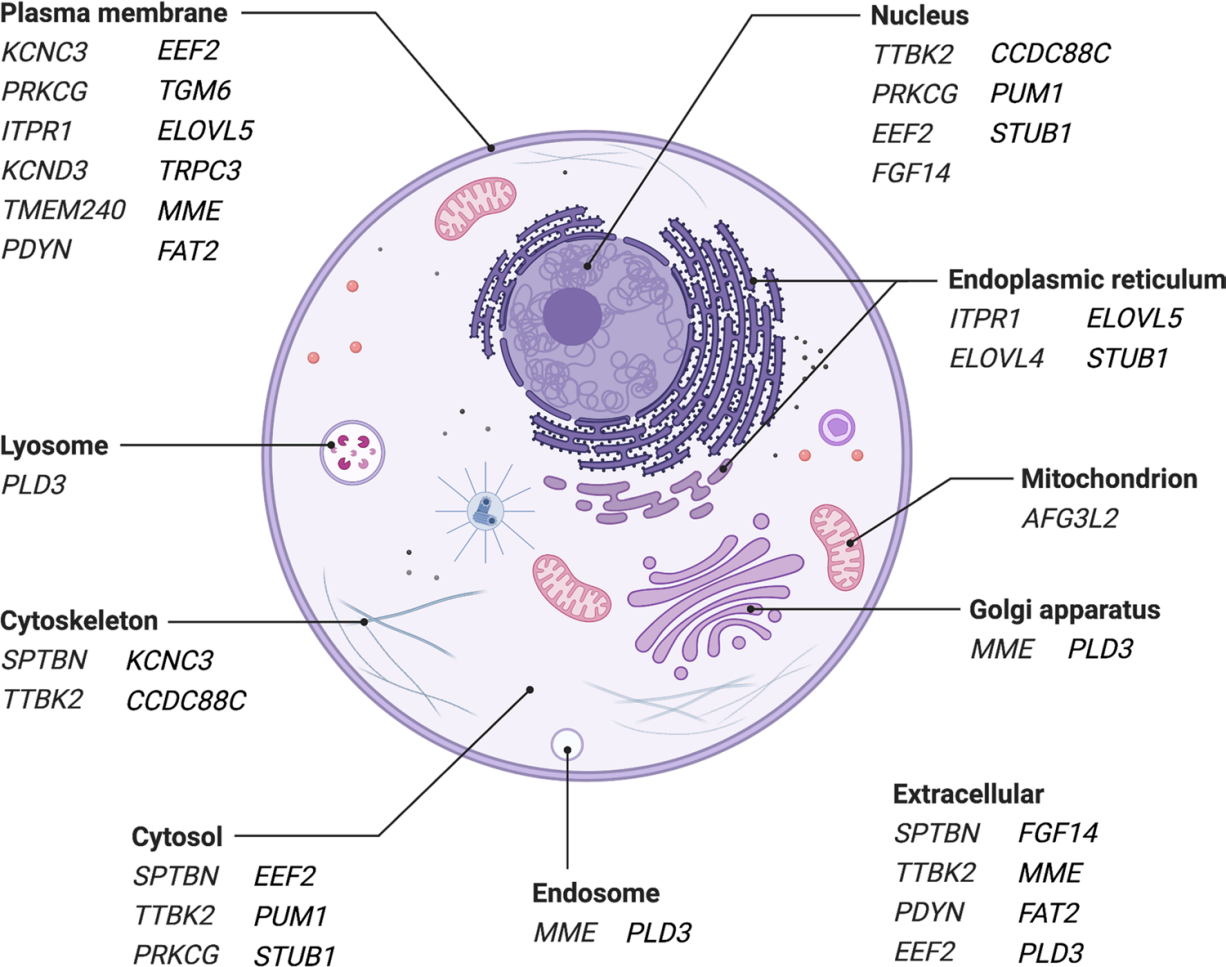
Subcellular location of polypeptides encoded by 21 genes involved in spinocerebellar degeneration. Note that one gene product can be present in more than one cell compartment. Only locations assigned with highest confidence (levels 4, 5 of genecards) are given. From: https://www.genecards.org/

The most striking pathological mechanism in the SCAs described is disturbance of calcium equilibrium in cells of patients. In fact, of the 21 disease genes in the SCAs discussed, the majority (Table 1) operates within or upstream of pathways that regulate intracellular Ca^2+^ levels. Significantly, many of the polypeptides encoded by the disease genes are located in cellular membranes (endoplasmic reticulum (ER) and cell plasma, Fig. 3). Alterations of these polypeptides due to mutations in the respective coding genes disturb integrity of the membranes. This can result in abnormal membrane permeability for various substances including Ca^2+^.

In most cases, no close interdependence of disease genes within a pathway has been identified. However, in two types of SCA, i.e., SCA14 and SCA41, the products of the disease genes *PRKCG* (encoding PKCγ) and *TRPC3* (encoding a transient receptor potential cation channel) closely interact in the maintenance of Ca^2+^ homeostasis (Fig. 1). Mutations in *TRPC3* compromise the function of the receptor thus reducing Ca^2+^ uptake by the cell. Conversely, mutations of *PRKCG* interfere with the negative PKCγ-mediated regulation of the receptor and make a larger than physiological amount of calcium enter the cell.

Disturbed homeostasis of Ca^2+^ affects important functions of neurons, such as the regulation of neurite outgrowth, synaptogenesis, synaptic transmission and plasticity, and cell survival [116, 117]. In several SCAs, gene mutations directly affect synapses. Another group of mutations interferes with maintenance and function of the cytoskeleton, which eventually results in cell death. The cytoskeleton is physiologically regulated by Ca^2+^. Therefore — similar to disturbed synapse development and function — the mutation might either affect the cytoskeleton directly or indirectly via disturbed Ca^2+^ homeostasis.

Other disease mechanisms include dysfunction of mitochondria either by mutations in nuclear (SCA28) or mitochondrial (adult-onset SCA, mitochondrial) genes. These mutations are thought to disturb mitochondrial energy production and thus might cause cell death. Additional possible disease mechanisms have been discussed above in the context of relevant SCAs.

In summary, the majority of SCAs discussed here is caused by mutations interfering with neuronal Ca^2+^ equilibrium. This eventually results in cell death, e.g., by interfering with development of synapses or the cytoskeleton.

## Data Availability

Not applicable. My manuscript has no associated data or the data will not be deposited.

## References

[1] Sullivan R, Yau WY, O’Connor E, Houlden H (2019). Spinocerebellar ataxia: an update. J Neurol.

[2] Buijsen RA, Toonen LJ, Gardiner SL, van Roon-Mom WMC (2019). Genetics, mechanisms, and therapeutic progress in polyglutamine spinocerebellar ataxias. Neurotherapeutics.

[3] Rasmussen A, Matsuura T, Ruano L, Yescas P, Ochoa A, Ashizawa T (2001). Clinical and genetic analysis of four Mexican families with spinocerebellar ataxia type 10. Ann Neurol.

[4] Seixas AI, Loureiro JR, Costa C, Ordonez-Ugalde A, Marcelino H, Oliveira CL (2017). (2017) A pentanucleotide ATTTC repeat insertion in the non-coding region of DAB1, mapping to SCA37, causes spinocerebellar ataxia. Am J Hum Genet.

[5] Kobayashi H, Koji A, Matsuura T, Ikeda Y, Hitomi T, Akechi Y (2011). Expansion of intronic GGCCTG hexanucleotide repeat in NOP56 causes SCA36, a type of spinocerebellar ataxia accompanied by motor neuron involvement. Am J Hum Genet.

[6] Ranum LPW, Schut LJ, Lundgren JK, Orr HT, Livingston DM (1994). Spinocerebellar ataxia type 5 in a family descended from the grandparents of President Lincoln maps to chromosome 11. Nature Genet.

[7] Stevanin G, Herman A, Brice A, Durr A (1999). Clinical and MRI findings in spinocerebellar ataxia type 5. Neurology.

[8] Schnekenberg RP, Perkins EM, Miller JW, Davies WIL, D'Adamo MC, Pessia M (2015). De novo point mutations in patients diagnosed with ataxic cerebral palsy. Brain.

[9] Bürk K, Zühlke C, König IR, Ziegler A, Schwinger E, Globas C (2004). Spinocerebellar ataxia type 5: clinical and molecular genetic features of a German kindred. Neurology.

[10] Ikeda Y, Dick KA, Weatherspoon MR, Gincel D, Armbrust KR, Dalton JC (2006). Spectrin mutations cause spinocerebellar ataxia type 5. Nat Genet.

[11] Bian X, Wang S, Jin S, Xu S, Zhang H, Wang D (2021). Two novel missense variants in SPTBN2 likely associated with spinocerebellar ataxia type 5. Neurol Sci.

[12] Jacob F-D, Ho ES, Martinez-Ojeda M, Darras BT, Khwaja OS (2012). Case of infantile onset spinocerebellar ataxia type 5. J Child Neurol.

[13] Nuovo S, Micalizzi A, D'Arrigo S, Ginevrino M, Biagini T, Mazza T (2018). Between SCA5 and SCAR14: delineation of the SPTBN2 p. R480W-associated phenotype. Eur J Hum Genet.

[14] Stankewich MC, Tse WT, Peters LL, Ch´ng Y, John KM, Stabach PR (1998). A widely expressed beta III spectrin associated with Golgi and cytoplasmic vesicles. Proc Natl Acad Sci USA.

[15] Eckley DM, Schroer TA (2003). Interactions between the evolutionarily conserved, actin-related protein, Arp11, Actin, and Arp1. Mol Biol Cell.

[16] Avery AW, Thomas DD, Hays TS (2017). β-III-spectrin spinocerebellar ataxia type 5 mutation reveals a dominant cytoskeletal mechanism that underlies dendritic arborization. Proc Natl Acad Sci USA.

[17] Worth PF, Giunti P, Gardner-Thorpe C, Dixon PH, Davis MB, Wood NW (1999). Autosomal dominant cerebellar ataxia type III: linkage in a large British family to a 7.6-cM region on chromosome 15q14-21.3. Am J Hum Genet.

[18] Houlden H, Johnson J, Gardner-Thorpe C, Lashley T, Hernandez D, Worth P (2007). Mutations in TTBK2, encoding a kinase implicated in tau phosphorylation, segregate with spinocerebellar ataxia type 11. Nature Genet.

[19] Bauer P, Stevanin G, Beetz C, Synofzik M, Schmitz-Hübsch T, Wüllner U (2010). Spinocerebellar ataxia type 11 (SCA11) is an uncommon cause of dominant ataxia among French and German kindreds. J Neurol Neurosurg Psychiatry.

[20] Bowie E, Norris R, Anderson KV, Goetz SC (2018). Spinocerebellar ataxia type 11-associated alleles of Ttbk2 dominantly interfere with ciliogenesis and cilium stability. PloS Genet.

[21] Takahashi M, Tomizawa K, Sato K, Ohtake A, Omori A (1995). A novel tau-tubulin kinase from bovine brain. FEBS Lett.

[22] Watanabe T, Kakeno M, Matsui T, Sugiyama I, Arimura N, Matsuzawa K (2015). TTBK2 with EB1/3 regulates microtubule dynamics in migrating cells through KIF2A phosphorylation. J Cell Biol.

[23] Jiang K, Toedt G, Montenegro Gouveia S, Davey NE, Hua S, van der Vaart B (2012). Proteome-wide screen for mammalian SxIP motif-containing microtubule plus-end tracking proteins. Curr Biol.

[24] Goetz SC, Liem KF, Anderson KV (2012). The spinocerebellar ataxia-associated gene Tau tubulin kinase 2 controls the initiation of ciliogenesis. Cell.

[25] Liao J-C, Yang TT, Weng RR, Kuo C-T, Chang C-W (2015) TTBK2: a Tau protein kinase beyond Tau phosphorylation. BioMed Research International Volume 2015 Article ID 575170, 10 pages 10.1155/2015/575170 10.1155/2015/575170 PMC4407412 25950000

[26] Herman-Bert A, Stevanin G, Netter J-C, Rascol O, Brassat D, Calvas P (2000). Mapping of spinocerebellar ataxia 13 to chromosome 19q13.3-q13.4 in a family with autosomal dominant cerebellar ataxia and mental retardation. Am J Hum Genet.

[27] Subramony SH, Advincula J, Perlman S, Rosales RL, Lee LV, Ashizawa T (2013). Waters, M. F. Comprehensive phenotype of the p.arg420his allelic form of spinocerebellar ataxia type 13. Cerebellum.

[28] Pyle A, Smertenko T, Bargiela D, Griffin H, Duff J, Appleton M (2015). Exome sequencing in undiagnosed inherited and sporadic ataxias. Brain.

[29] Waters MF, Fee D, Figueroa KP, Nolte D, Muller U, Advincula J (2005). An autosomal dominant ataxia maps to 19q13: allelic heterogeneity of SCA13 or novel locus?. Neurology.

[30] Waters MF, Minassian NA, Stevanin G, Figueroa KP, Bannister JPA, Nolte D (2006). Mutations in voltage-gated potassium channel KCNC3 cause degenerative and developmental nervous system phenotypes. Nature Genet.

[31] Duarri A, Nibbeling EAR, Fokkens MR, Meijer M, Boerrigter M, Verschuuren-Bemelmans CC (2015). Functional analysis helps to define KCNC3 mutational spectrum in Dutch ataxia cases. PLoS ONE.

[32] Zhang GY, Zhang XF, Fleming MR, Amiri A, El-Hassar L, Surguchev AA (2016). Kv3.3 channels bind Hax-1 and Arp2/3 to assemble a stable local actin network that regulates channel gating. Cell.

[33] Zhao J, Zhu J, Thornhill WB (2013). Spinocerebellar ataxia-13 Kv3.3 potassium channels: arginine-to-histidine mutations affect both functional and protein expression on the cell surface. Biochem J.

[34] Yamashita I, Sasaki H, Yabe I, Fukazawa T, Nogoshi S, Komeichi K (2000). A novel locus for dominant cerebellar ataxia (SCA14) maps to a 10.2-cM interval flanked by D19S206 and D19S605 on chromosome 19q13.4-qter. Ann Neurol.

[35] Stevanin G, Hahn V, Lohmann E, Bouslam N, Gouttard M, Soumphonphakdy C (2004). Mutation in the catalytic domain of protein kinase C gamma and extension of the phenotype associated with spinocerebellar ataxia type 14. Arch Neurol.

[36] Klebe S, Durr A, Rentschler A, Hahn-Barma V, Abele M, Bouslam N (2005). New mutations in protein kinase C gamma associated with spinocerebellar ataxia type 14. Ann Neurol.

[37] Chen D-H, Brkanac Z, Verlinde CLMJ, Tan X-J, Bylenok L, Nochlin D et al (2003) Missense mutations in the regulatory domain of PKC-gamma: a new mechanism for dominant nonepisodic cerebellar ataxia. Am J Hum Genet 72:839–849. 0002–9297/2003/7204–0007 10.1086/373883 PMC1180348 12644968

[38] Brkanac Z, Bylenok L, Fernandez M, Matsushita M, Lipe H, Wolff J (2002). A new dominant spinocerebellar ataxia linked to chromosome 19q13.4-qter. Arch Neurol.

[39] Wong MMK, Hoekstra SD, Vowles J, Watson LM, Fuller G, Nemeth AH (2018). Neurodegeneration in SCA14 is associated with increased PKCγ kinase activity, mislocalization and aggregation. Acta neuropathol commun.

[40] Alonso I, Costa C, Gomes A, Ferro A, Seixas AI, Silva S (2005). A novel H101Q mutation causes PKC-gamma loss in spinocerebellar ataxia type 14. J Hum Genet.

[41] Najmabadi H, Hu H, Garshasbi M, Zemojtel T, Abedini SS, Chen W (2011). Deep sequencing reveals 50 novel genes for recessive cognitive disorders. Nature.

[42] Saito S, Protein SY (2002). Kinase Cγ (PKCγ): function of neuron specific isotype. J Biochem.

[43] Meier J, Akyeli J, Kirischuk S, Grantyn RGABA(A),  (2003). Receptor activity and PKC control inhibitory synaptogenesis in CNS tissue slices. Mol Cell Neurosci.

[44] Garzón-Niño J, Rodríguez-Muñoz M, Cortés-Montero E, Sánchez-Blázquez P (2017). Increased PKC activity and altered GSK3β/NMDAR function drive behavior cycling in HINT1-deficient mice: bipolarity or opposing forces. Sci Rep.

[45] Storey E, Gardner RJM, Knight MA, Kennerson ML, Tuck RR, Forrest SM (2001). A new autosomal dominant pure cerebellar ataxia. Neurology.

[46] Miura S, Shibata H, Furuya H, Ohyagi Y, Osoegawa M, Miyoshi Y (2006). The contactin 4 gene locus at 3p26 is a candidate gene of SCA16. Neurology.

[47] Synofzik M, Beetz C, Bauer C, Bonin M, Sanchez-Ferrero E, Schmitz-Hubsch T (2011). Spinocerebellar ataxia type 15: diagnostic assessment, frequency, and phenotypic features. J Med Genet.

[48] Knight MA, Kennerson ML, Anney RJ, Matsuura T, Nicholson GA, Salimi-Tari P (2003). Spinocerebellar ataxia type 15 (SCA15) maps to 3p24.2-3pter: exclusion of the ITPR1 gene, the human orthologue of an ataxic mouse mutant. Neurobiol Dis.

[49] Hara K, Fukushima T, Suzuki T, Shimohata T, Oyake M, Ishiguro H (2004). Japanese SCA families with an unusual phenotype linked to a locus overlapping with SCA15 locus. Neurology.

[50] van de Leemput J, Chandran J, Knight MA, Holtzclaw LA, Scholz S, Cookson MR (2007). Deletion at ITPR1 underlies ataxia in mice and spinocerebellar ataxia 15 in humans. PLoS Genet.

[51] Hara K, Shiga A, Nozaki H, Mitsui J, Takahashi Y, Ishiguro H (2008). Total deletion and a missense mutation of ITPR1 in Japanese SCA15 families. Neurology.

[52] Marelli C, van de Leemput J, Johnson JO, Tison F, Thauvin-Robinet C, Picard F (2011). SCA15 due to large ITPR1 deletions in a cohort of 333 white families with dominant ataxia. Arch Neurol.

[53] Kass GE, Orrenius S (1999). Calcium signaling and cytotoxicity. Environ Health Perspect.

[54] Kasumu A, Bezprozvanny I (2012). Deranged calcium signaling in Purkinje cells and pathogenesis in spinocerebellar ataxia 2 (SCA2) and other ataxias. Cerebellum.

[55] Brkanac Z, Fernandez M, Matsushita M, Lipe H, Wolff J, Bird T (2002). Autosomal dominant sensory/motor neuropathy with ataxia (SMNA): linkage to chromosome 7q22-q32. Am J Med Genet.

[56] Brkanac Z, Spencer D, Shendure J, Robertson RD, Matsushita M, Vu T (2009). IFRD1 is a candidate gene for SMNA on chromosome 7q22-q23. Am J Hum Genet.

[57] Schelhaas HJ, Ippel PF, Hageman G, Sinke RJ, van der Laan EN, Beemer FA (2001). Clinical and genetic analysis of a four-generation family with a distinct autosomal dominant cerebellar ataxia. J Neurol.

[58] Lee Y-C, Durr A, Majczenko K, Huang Y-H, Liu Y-C, Lien C-C (2012). Mutations in KCND3 cause spinocerebellar ataxia type 22. Ann Neurol.

[59] Huin V, Strubi-Vuillaume I, Dujardin K, Brion M, Delliaux M (2017). Dellacherie D et al Expanding the phenotype of SCA 19/22: parkinsonism, cognitive impairment and epilepsy. Parkinsonism Relat Disord.

[60] Duarri A, Jezierska J, Fokkens M, Meijer M, Schelhaas HJ, den Dunnen WFA (2012). Mutations in potassium channel KCND3 cause spinocerebellar ataxia type 19. Ann Neurol.

[61] Verbeek DS, Schelhaas JH, Ippel EF, Beemer FA, Pearson PL, Sinke RJ (2002). Identification of a novel SCA locus (SCA19) in a Dutch autosomal dominant cerebellar ataxia family on chromosome region 1p21-q21. Hum Genet.

[62] Chung M, Lu Y-C, Cheng N-C, Soong B-W (2003). A novel autosomal dominant spinocerebellar ataxia (SCA22) linked to chromosome 1p21-q23. Brain.

[63] Ohya S, Tanaka M, Oku T, Asai Y, Watanabe M, Giles WR (1997). Molecular cloning and tissue distribution of an alternativelyspliced variant of an A-type K1channel alpha-subunit, Kv4.3 in the rat. FEBS Lett.

[64] Serodio P, Rudy B (1998). Differential expression of Kv4 K1channel subunitsmediating subthreshold transient K1(A-type) current s in rat brain. J Neurophysiol.

[65] Knight MA, Gardner RJ, Bahlo M, Matsuura T, Dixon JA, Forrest SM (2004). Dominantly inherited ataxia and dysphonia with dentate calcification: spinocerebellar ataxia type 20. Brain.

[66] Knight MA, Hernandez D, Diede SJ, Dauwerse HG, Rafferty I, van de Leemput J (2008). A duplication at chromosome 11q12.2-11q12.3 is associated with spinocerebellar ataxia type 20. Hum Mol Genet.

[67] Devos D, Schraen-Maschke S, Vuillaume I, Dujardin K, Naze P, Willoteaux C (2001). (2001) Clinical features and genetic analysis of a new form of spinocerebellar ataxia. Neurology.

[68] Delplanque J, Devos D, Huin V, Genet A, Sand O, Moreau C (2014). TMEM240 mutations cause spinocerebellar ataxia 21 with mental retardation and severe cognitive impairment. Brain.

[69] Hawrylycz M, Lein E, Guillozet-Bongaarts A (2012). An anatomically comprehensive atlas of the adult human brain transcriptome. Nature.

[70] Trinidad JC, Barkan DT, Gulledge BF, Thalhammer A, Sali A, Schoepfer R (2012). Global identification and characterization of both O-GlcNAcylation and phosphorylation at the murine synapse. Mol Cell Proteomics.

[71] Verbeek DS, van de Warrenburg BP, Wesseling P, Pearson PL, Kremer HP, Sinke RJ (2004). Mapping of theSCA23 locus involved in autosomal dominant cerebellar ataxia to chromosome region 20p13-12.3. Brain.

[72] Bakalkin G, Watanabe H, Jezierska J, Depoorter C, Verschuuren-Bemelmans C, Bazov I (2010). Prodynorphin mutations cause the neurodegenerativedisorder spinocerebellar ataxia type 23. Am J Hum Genet.

[73] Hauser KF, Aldrich JV, Anderson KJ, Bakalkin G, Christie MJ, Hall ED (2005). Pathobiology of dynorphins in trauma and disease. Front Biosci.

[74] Schadrack J, Willoch F, Platzer S, Bartenstein P, Mahal B, Dworzak D (1999). Opioid receptors in the human cerebellum: evidence from [11C]diprenorphine PET, mRNA expression and autoradiography. NeuroReport.

[75] Stevanin G, Bouslam N, Thobois S, Azzedine H, Ravaux L, Boland A (2004). Spinocerebellar ataxia with sensory neuropathy (SCA25) maps to chromosome 2p. Ann Neurol.

[76] Yu G-Y, Howell MJ, Roller MJ, Xie T-D, Gomez CM (2005). Spinocerebellar ataxia type 26 maps to chromosome 19p13.3 adjacent to SCA6. Ann Neurol.

[77] Ortiz PA, Kinzy TG (2005). Dominant-negative mutant phenotypes and the regulation of translation elongation factor 2 levels in yeast. Nucleic Acids Res.

[78] Susorov D, Zakharov N, Shuvalova E, Ivanov A, Egorova T, Shuvalov A (2018). Eukaryotic translation elongation factor 2 (eEF2) catalyzes reverse translocation of the eukaryotic ribosome. J biol Chem.

[79] Hekman E, Yu G-Y, Brown CD, Zhu H, Du X, Gervin K (2012). A conserved eEF2 coding variant in SCA26 leads to loss of translational fidelity and increased susceptibility to proteostatic insult. Hum Mol Genet.

[80] van Swieten JC, Brusse E, de Graaf BM, Krieger E, van de Graaf R, de Koning I (2003). A mutation in the fibroblast growth factor 14 gene is associated with autosomal dominant cerebellar ataxia. Am J Hum Genet.

[81] Dalski A, Atici J, Kreuz FR, Hellenbroich Y, Schwinger E, Zuhlke C (2005). Mutation analysis in the fibroblast growth factor 14 gene: frameshift mutation and polymorphisms in patients with inherited ataxias. Eur J Hum Genet.

[82] Miura S, Kosaka K, Fujioka R, Uchiyama Y, Shimojo T, Morikawa T (2019). Spinocerebellar ataxia 27 with a novel nonsense variant (lys177X) in FGF14. Eur J Med Genet.

[83] Smallwood PM, Munoz-Sanjuan I, Tong P, Macke JP, Hendry SH, Gilbert DJ (1996). Fibroblast growth factor (FGF) homologous factors: new members of the FGF family implicated in nervous system development. Proc Natl Acad Sci USA.

[84] Cagnoli C, Mariotti C, Taroni F, Seri M, Brussino A, Michielotto C (2006). SCA28, a novel form of autosomal dominant cerebellar ataxia on chromosome 18p11.22-q11.2. Brain.

[85] Cagnoli C, Stevanin G, Brussino A, Barberis M, Mancini C, Margolis RL (2010). Missense mutations in the AFG3L2 proteolytic domain account for ~1.5% of European autosomal dominant cerebellar ataxias. Hum Mutat.

[86] Di Bella D, Lazzaro F, Brusco A, Plumari M, Battaglia G, Pastore A (2010). Mutations in the mitochondrial protease gene AFG3L2 cause dominant hereditary ataxia SCA28. Nature Genet.

[87] Storey E, Bahlo M, Fahey MC, Sisson O, Lueck LJ, Gardner RJM (2008). A new dominantly inherited pure cerebellar ataxia, SCA 30. J Neurol Neurosurg Psychiatry.

[88] Jiang H, Zhu, H-P, Gomez CM (2010) SCA32: an autosomal dominant cerebellar ataxia with azoospermia maps to chromosome 7q32-q33. (Abstract) Mov Disord 25: S192

[89] Cadieux-Dion M, Turcotte-Gauthier M, Noreau A, Martin C, Meloche C, Gravel M (2014). Expanding the clinical phenotype associated with ELOVL4 mutation: study of a large French-Canadian family with autosomal dominant spinocerebellar ataxia and erythrokeratodermia. JAMA Neurol.

[90] Ozaki K, Doi H, Mitsui J, Sato N, Iikuni Y, Majima T (2015). A novel mutation in ELOVL4 leading to spinocerebellar ataxia (SCA) with the hot cross bun sign but lacking erythrokeratodermia: a broadened spectrum of SCA34. JAMA Neurol.

[91] Giroux J-M, Barbeau A (1972). Erythrokeratodermia with ataxia. Arch Derm.

[92] Wang JL, Yang X, Xia K, Hu ZM, Weng L, Jin X (2010). TGM6 identified as a novel causative gene of spinocerebellar ataxias using exome sequencing. Brain.

[93] Li M, Pang SY, Song Y, Kung MHW, Ho S-L, Sham P-C (2013). Whole exome sequencing identifies a novel mutation in the transglutaminase 6 gene for spinocerebellar ataxia in a Chinese family. Clin Genet.

[94] Guo Y-C, Lin J-J, Liao Y-C, Tsai P-C, Lee Y-C, Soong B-W (2014). Spinocerebellar ataxia 35: novel mutations in TGM6 with clinical and genetic characterization. Neurology.

[95] Di Gregorio E, Borroni B, Giorgio E, Lacerenza D, Ferrero M, Lo Buono N (2014). ELOVL5 mutations cause spinocerebellar ataxia 38. Am J Hum Genet.

[96] Rizzuto R, Pozzan T (2006). Microdomains of intracellular Ca^2+^: molecular determinants and functional consequences. Physiol Rev.

[97] Johnson JO, Stevanin G, van de Leemput J, Hernandez DG, Arepalli S, Forlani S (2015). A 7.5-Mb duplication at chromosome 11q21-11q22.3 is associated with a novel spastic ataxia syndrome. Mov Disord.

[98] Tsoi H, Yu ACS, Chen ZS, Ng NKN, Chan AYY, Yuen LYP (2014). A novel missense mutation in CCDC88C activates the JNK pathway and causes a dominant form of spinocerebellar ataxia. J Med Genet.

[99] Fogel BL, Hanson SM, Becker EB (2015). Do mutations in the murine ataxia gene TRPC3 cause cerebellar ataxia in humans?. Mov Disord.

[100] Becker EB (2014). The Moonwalker mouse: new insights into TRPC3 function, cerebellar development, and ataxia. Cerebellum.

[101] Depondt C, Donatello S, Rai M, Wang FC, Manto M, Simonis N (2016). MME mutation in dominant spinocerebellar ataxia with neuropathy (SCA43). Neurol Genet.

[102] Nibbeling EAR, Duarri A, Verschuuren-Bemelmans CC, Fokkens MR, Karjalainen JM, Smeets CJLM (2017). (2017) Exome sequencing and network analysis identifies shared mechanisms underlying spinocerebellar ataxia. Brain.

[103] Tonholo Silva TY, Rosa ABR, Quaio CR, Verbeek D, Luiz Pedroso JL, Barsottini O (2021). Does SCA45 cause very late-onset pure cerebellar ataxia?. Neurol Genet.

[104] Nakayama M, Nakjima D, Yoshimura R, Endo Y, Ohara O (2002). MEGF1/fat2 proteins containing extraordinarily large extracellular domains are localized to thin parallel fibers of cerebellar granule cells. Mol Cell Neurosci.

[105] Gennarino VA, Palmer EE, McDonell LM, Wang L, Adamski CJ, Koire A (2018). A mild PUM1 mutation is associated with adult-onset ataxia, whereas haploinsufficiency causes developmental delay and seizures. Cell.

[106] Wilson BT, Omer M, Hellens SW, Zwolinski SA, Yates LM, Lynch SA (2015). Microdeletion 1p35.2: a recognizable facial phenotype with developmental delay. Am J Med Genet A.

[107] Gennarino VA, Singh RK, White JJ, De Maio A, Han K, Kim J-Y (2015). Pumilio1 haploinsufficiency leads to SCA1-like neurodegeneration by increasing wild-type ataxin1 levels. Cell.

[108] Kazantsev A, Preisinger E, Drankovsky A, Goldgaber D, Housman D (1999). Insoluble detergent-resistant aggregates form betweenpathological and nonpathological lengths of polyglutaminein mammalian cells. Proc Natl Acad Sci USA.

[109] De Michele G, Lieto M, Galatolo D, Salvatore E, Cocozza S, Barghigiani M (2019). Spinocerebellar ataxia 48 presenting with ataxia associated with cognitive, psychiatric, and extrapyramidal features: a report of two Italian families. Parkinsonism Relat Disord.

[110] Genis D, Ortega-Cubero S, San Nicolás H, Corral J, Gardenyes J, de Jorge L, López E (2018). Heterozygous STUB1 mutation causes familial ataxia with cognitive affective syndrome (SCA48). Neurology.

[111] Palvadeau R, Kaya-Gulec ZE, Simsir G, Vural A, Oztop-Cakmak O, Genc G (2020). Cerebellar cognitive-affective syndrome preceding ataxia associated with complex extrapyramidal features in a Turkish SCA48 family. Neurogenetics.

[112] Pfeffer G, Blakely EL, Aöston CL, Hassani A, Boggild M, Jorvath R (2012). Adult-onset spinocerebellar ataxia syndromes due to MTATP6 mutations. J Neurol Neurosurg Psychiatry.

[113] Nolte D, Kang J-S, Hofmann A, Schwaab E, Krämer HH, Müller U (2021). Mutations in MT-ATP6 are a frequent cause of adult-onset spinocerebellar ataxia. J Neurol.

[114] Ganetzky RD, Stendel C, McCormick EM, Zolkipli-Cunningham Z (2019). MT-ATP6 mitochondrial disease variants: phenotypic and biochemical features analysis in 218 published cases and cohort of 14 new cases. Hum Mutat.

[115] Paulson HL, Bonini NM, Roth KA (2000). Polyglutamine disease and neuronal cell death. Proc Natl Acad Sci.

[116] Mattson MP (2007). Calcium and neurodegeneration. Aging Cell.

[117] Gasperinia RJ, Paveza M, Thompsona AC, Mitchell CB (2017). How does calcium interact with the cytoskeleton to regulate growth cone motility during axon pathfinding?. Mol Cell Neurosci.

[118] Trebak M (2010). The puzzling role of TRPC3 channels in motor coordination. Pflugers Arch.

